# Transdiagnostic Risk Calculator for the Automatic Detection of Individuals at Risk and the Prediction of Psychosis: Second Replication in an Independent National Health Service Trust

**DOI:** 10.1093/schbul/sby070

**Published:** 2018-06-12

**Authors:** Paolo Fusar-Poli, Nomi Werbeloff, Grazia Rutigliano, Dominic Oliver, Cathy Davies, Daniel Stahl, Philip McGuire, David Osborn

**Affiliations:** 1 Early Psychosis: Interventions and Clinical Detection (EPIC) Lab, Department of Psychosis Studies, Institute of Psychiatry, Psychology & Neuroscience, King’s College London, London, UK; 2 OASIS Service, South London and Maudsley NHS Foundation Trust, London, UK; 3 Department of Brain and Behavioral Sciences, University of Pavia, Pavia, Italy; 4 Division of Psychiatry, University College London, London, UK; 5 Camden and Islington NHS Foundation Trust, London, UK; 6 Department of Biostatistics, Institute of Psychiatry, Psychology & Neuroscience, King’s College London, London, UK; 7 Department of Psychosis Studies, Institute of Psychiatry, Psychology & Neuroscience, King’s College London, London, UK

**Keywords:** psychosis, schizophrenia, transdiagnostic, risk calculator, validation

## Abstract

**Background:**

The benefits of indicated primary prevention among individuals at Clinical High Risk for Psychosis (CHR-P) are limited by the difficulty in detecting these individuals. To overcome this problem, a transdiagnostic, clinically based, individualized risk calculator has recently been developed and subjected to a first external validation in 2 different catchment areas of the South London and Maudsley (SLaM) NHS Trust.

**Methods:**

Second external validation of real world, real-time electronic clinical register-based cohort study. All individuals who received a first ICD-10 index diagnosis of nonorganic and nonpsychotic mental disorder within the Camden and Islington (C&I) NHS Trust between 2009 and 2016 were included. The model previously validated included age, gender, ethnicity, age by gender, and ICD-10 index diagnosis to predict the development of any ICD-10 nonorganic psychosis. The model’s performance was measured using Harrell’s C-index.

**Results:**

This study included a total of 13702 patients with an average age of 40 (range 16–99), 52% were female, and most were of white ethnicity (64%). There were no CHR-P or child/adolescent services in the C&I Trust. The C&I and SLaM Trust samples also differed significantly in terms of age, gender, ethnicity, and distribution of index diagnosis. Despite these significant differences, the original model retained an acceptable predictive performance (Harrell’s C of 0.73), which is comparable to that of CHR-P tools currently recommended for clinical use.

**Conclusions:**

This risk calculator may pragmatically support an improved transdiagnostic detection of at-risk individuals and psychosis prediction even in NHS Trusts in the United Kingdom where CHR-P services are not provided.

## Introduction

Individuals at Clinical High Risk for Psychosis (CHR-P^1^) present with subtle symptoms and overall functional impairment.^2^ In the wake of these problems, they seek help at specialized CHR-P clinics.^3^ CHR-P individuals have 20% probability of developing incident psychotic disorders (but not other nonpsychotic disorders^4,5^) over a relatively short period of 2 years.^6^ Primary indicated prevention in CHR-P individuals has the unique potential to alter the course of psychosis^7^ and reduce the duration of untreated psychosis,^8,9^ while secondary prevention in CHR-P who will develop the disorder can ameliorate the severity of the first episode.^10^

**Fig. 1.**
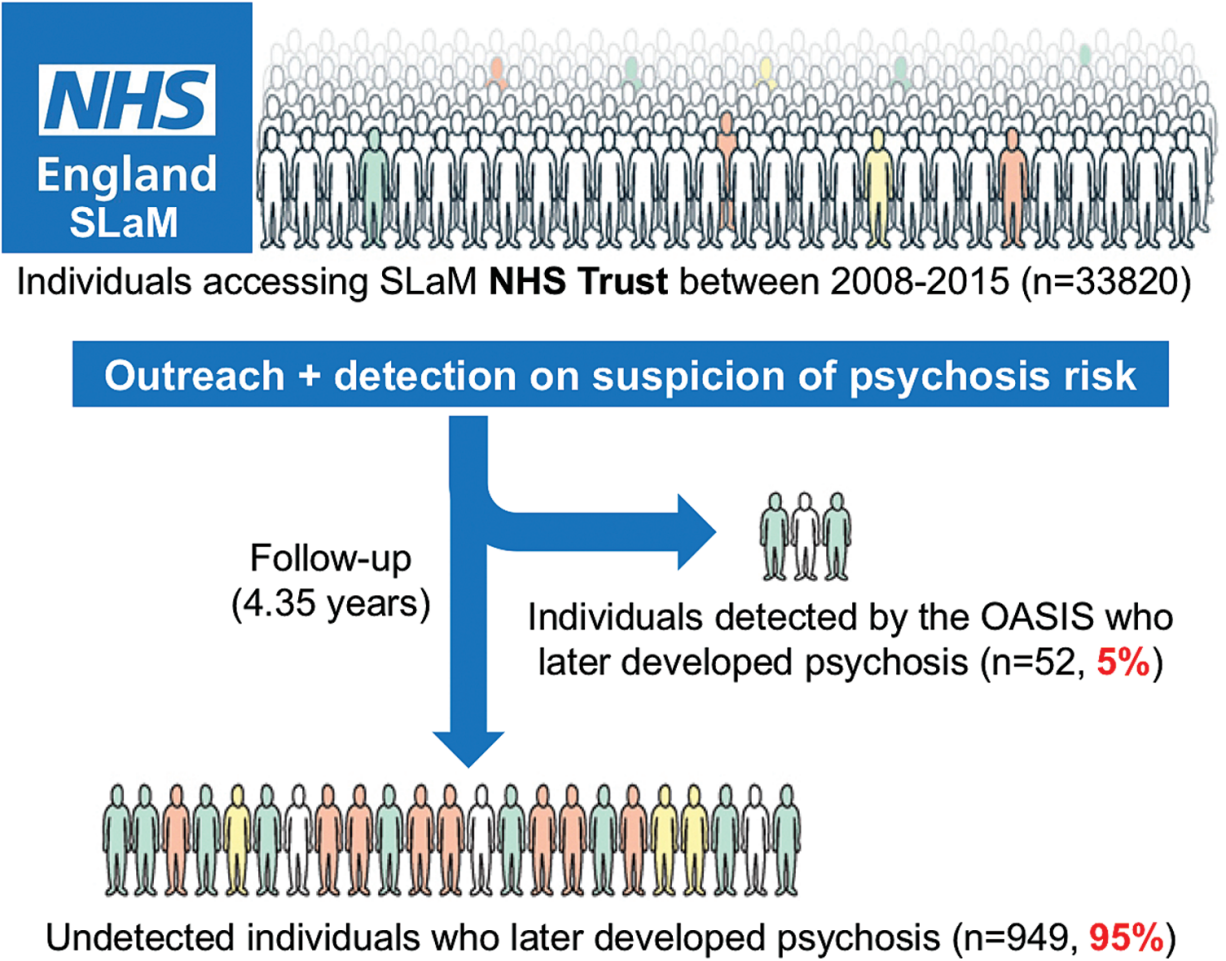
Current detection strategies for individuals at risk of psychosis in secondary mental health care in South London, UK. The local early detection service (OASIS^3^), which is embedded in the South London and Maudsley (SLaM) NHS Trust, runs an ongoing outreach to promote referrals on suspicion of psychosis risk.^11^ This strategy is highly inefficient and misses 95% of patients who are at risk and who will develop a first episode of psychosis over the ensuing 4 years.^12^

**Fig. 2.**
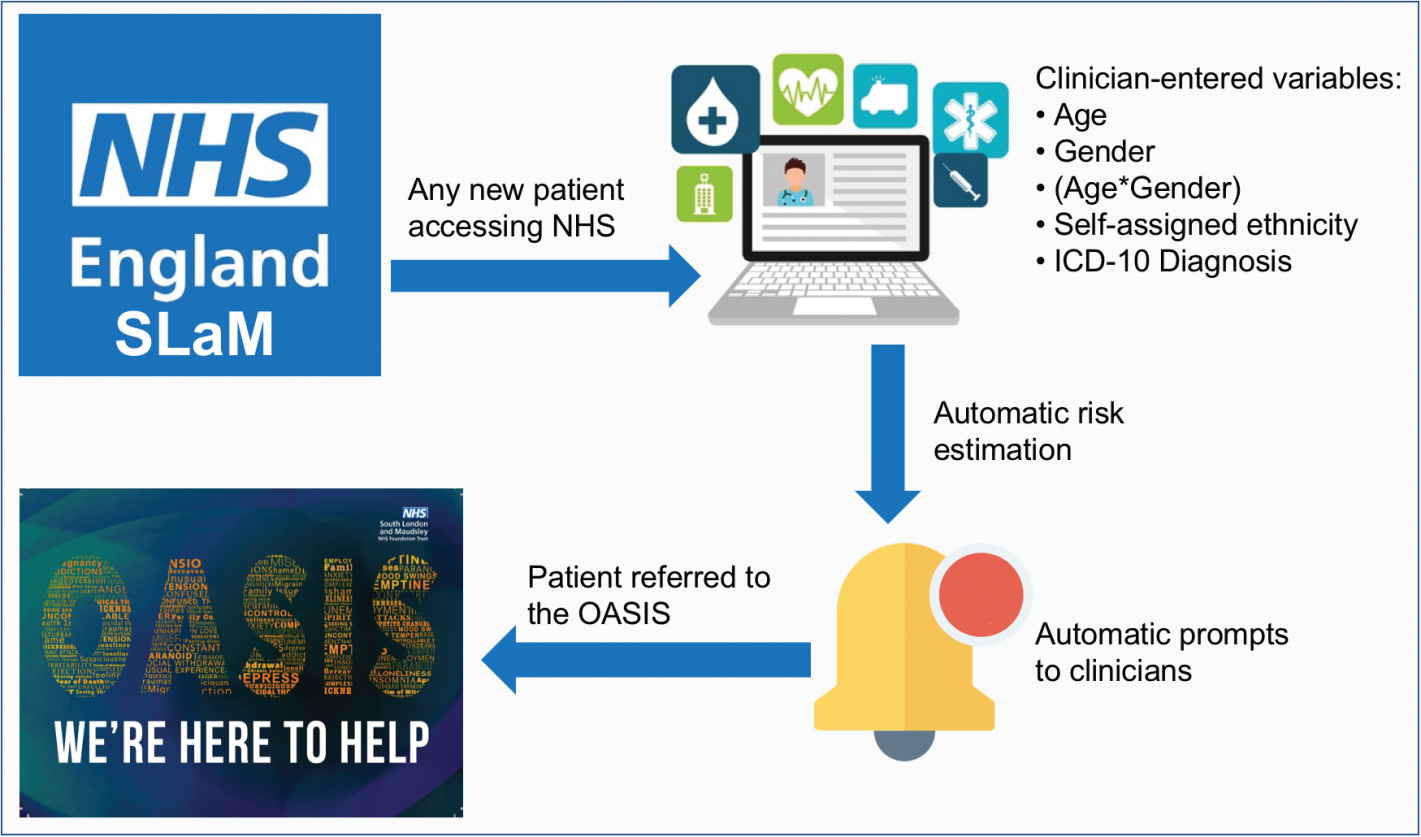
Potential clinical use of the individualized, clinically based, transdiagnostic risk calculator in secondary mental health care. For any new patient accessing the local NHS Trust (South London and Maudsley, UK) clinicians will enter the predictors on the electronic case register, as part of their clinical routine. The calculator, embedded in the electronic system, would then use the predictors to estimate the individual risk of developing psychosis over time. This information would then be shared with clinicians through automated alerts, inform their decision making and promote appropriate referrals to the local early detection clinic (OASIS^3^).

Currently, these benefits are offered only to those CHR-P individuals who are detected by existing recruitment strategies, which rely on: (1) outreach campaigns and (2) referrals made on suspicion of psychosis risk (figure 1). These strategies are highly inefficient.^12^ A recent study estimated that of the 1001 first-episode cases in secondary mental health care within the South London and Maudsley (SLaM) NHS Trust, only 52 (5%) were referred to, and therefore detected by, the local CHR-P service before the manifestation of their illness^13^ (figure 1). The negligible number of cases detected in real-life settings by the CHR-P paradigm is highly problematic because it severely hinders our ability to change the course of psychosis.^12^ Furthermore, there is a window of missed opportunity for the prevention of psychosis because all of these individuals were already under the care of mental health services.^13^ Leading experts have capitalized on these caveats to question the overall clinical utility of the CHR-P paradigm.^14^ To overcome these problems, a transdiagnostic, clinically based, individualized risk calculator has recently been developed in the Lambeth and Southwark boroughs of SLaM.^13^ The calculator employs electronic health data and therefore can be applied at scale to detect all individuals at risk of developing psychosis in secondary mental health care (see figure 2 for a simple presentation of this calculator). The model was developed following state-of-the-art guidelines which recommend preselecting predictors on the basis of a priori knowledge.^15^ Predictors were also limited in number to ensure that there was an adequate event per variable ratio (which is recommended to develop robust models in the case of relatively infrequent outcomes^16^) on the basis of an established a priori empirical link to psychosis risk: age,^17^ gender,^17^ ethnicity,^17^ and age by gender interaction.^17^ The ICD-10 nonpsychotic diagnoses were selected on the basis of evidence indicating that psychosis may emerge from several diagnostic spectra.^18^ The transdiagnostic properties of this tool mean that it can be used in a pragmatic fashion, not merely in CHR-P samples, but also in any patient receiving a first ICD-10 index diagnosis of any nonpsychotic mental disorder.^13^ This calculator has already demonstrated adequate prognostic performance in the first external validation in the SLaM boroughs of Lewisham and Croydon (Harrell’s C = 0.79, for full details see Fusar-Poli et al^13^). However, to date, the extent to which the calculator can be “transported” into other NHS Trusts—that may be characterized by different service configurations or patient sociodemographic and clinical characteristics—remains unknown. External validation studies are essential to evaluate the generalizability of predictive risk models and to assess whether they should be implemented in clinical practice.^19^

The current study investigates the external validity of the original transdiagnostic, clinically based, individualized risk calculator in an independent data set representative of the Camden and Islington (C&I) NHS Trust.

## Materials and Methods

### Setting: Camden and Islington NHS Trust and Clinical Record Interactive Search

C&I NHS Trust is a large mental health care provider that serves a geographic catchment area of 2 inner-city London boroughs, and nearly 470000 residents. It provides mental health and substance misuse services to adults living in Camden and Islington. In addition, the Trust provides substance misuse services to Westminster, as well as substance misuse and psychological therapies services to the residents of Kingston. It has 2 inpatient facilities, at Highgate Mental Health Centre and St Pancras Hospital, as well as community-based services throughout the London boroughs of Camden and Islington. The services accommodate adults of working age, adults with learning difficulties, and older people across the community or in inpatient settings. The Trust does not provide CHR-P services or child and adolescent services, with the exception of the Camden Early Intervention Service, which accepts referrals over the age of 14. The C&I Trust uses the same Clinical Record Interactive Search (CRIS) system as used in SLaM.^20^ The CRIS C&I database comprised complete but anonymized information from over 116000 mental health patients.^21^ C&I CRIS received ethical approval from the NRES Committee East of England—Cambridge Central (14/EE/0177).

### Statistical Analysis

This clinical register-based cohort study was conducted according to the REporting of studies Conducted using Observational Routinely collected health Data (RECORD) Statement^22^ (see checklist reported in supplementary eTable 1).

Baseline clinical and sociodemographic characteristics of the sample (including missing data) were described by means and SDs for continuous variables, and absolute and relative frequencies for categorical variables. The overall cumulative risk of psychosis onset (see below) in the C&I NHS Trust was described with the Kaplan–Meier failure function (1—survival)^23^ and Greenwood 95% CIs.^24^

#### Model Specifications.

We used the original transdiagnostic clinically based individualized risk calculator, which was developed through CRIS in the SLaM boroughs of Lambeth and Southwark, and validated in the SLaM boroughs of Croydon and Lewisham.^13^ In summary, a Cox model was used to predict as primary outcome of interest the hazard ratio (HR) of developing any psychotic disorder over time (see supplementary eMethods 1 for definition). The predictors included age (at the time of the index diagnosis), gender, age by gender, self-assigned ethnicity, and index diagnosis. Self-assigned ethnicity and index diagnoses were operationalized as indicated in supplementary eTables 2 and 3. These definitions matched the operationalizations of the original model.^13^ The follow-up commenced 3 months after the date of the index diagnosis within the C&I NHS Trust and was censored on October 30, 2016, in line with the previous analysis.^13^ As for the original report,^13^ this lag period was chosen to allow patients sufficient time after their index diagnosis to meet the ICD-10 duration criterion for brief psychotic episodes—that are usually included under the CHR-P designation^25^—and to exclude individuals who were underreporting psychotic symptoms at baseline (false transition to psychosis).^26^

#### Second External Model Validation.

Model validation followed the guidelines of Royston and Altman,^27^ Steyerberg et al,^28^ and the Transparent Reporting of a multivariable prediction model for Individual Prognosis Or Diagnosis (TRIPOD).^29^ An independent researcher (N.W.) who had access to the original data performed the analyses under the supervision of a senior researcher (P.F.P.) who also led the development and validation of the original risk calculator. To ensure proper external validation, P.F.P. limited his contribution to the sharing of the STATA script (supplementary eMethods 2). P.F.P. did not have any direct access to the original database and did not run the analyses.

To interpret the performance of a risk model in the context of external validation, it is essential to first quantify the degree of relatedness between development and validation samples.^19^ External validity only assesses model transportability if validation samples have a different case mix. Thus, we investigated the extent to which the SLaM and C&I data sets comprised patients with sets of prognostically relevant predictors in common, comparable time-to-event outcomes with roughly similar follow-up times, and the same clinical condition observed in similar settings.^30^ As a first step, we visually compared the 2 Kaplan–Meier failure functions. If the curves vary noticeably or if there are differences in their shapes, systematic differences within the study populations may be indicated.^30^ We also reported the spread (SD) and mean of the prognostic index of the model in the 2 NHS Trusts. An increased (or decreased) variability of the prognostic index would indicate more (or less) heterogeneity of case mix between the 2 NHS Trusts, and therefore, of their overarching target populations.^19^ Differences in the mean prognostic index indicate differences in overall (predicted) outcome frequency, reflecting case-mix severity between the 2 NHS Trusts (and revealing the model’s calibration-in-the-large in the C&I database).^19^ As a second step, we compared the distributions of predictors between the SLaM and C&I datasets.^30^ Here, substantial differences may also indicate important differences in the study populations.^30^

We then conducted the formal external validation.^30^ Accordingly, the regression coefficients estimated in the SLaM NHS Trust^13^ were applied to each case in the external C&I NHS Trust, to generate the prognostic index in the C&I NHS Trust. Since we were interested in discrimination, the primary outcome measure for model performance (accurate predictions discriminate between those with and those without the outcome^28^) was Harrell’s C-index.^27^ Harrell’s C is a recommended measure for external validation of Cox models according to established guidelines.^27^ Harrell’s C is the probability that for a random pair of “case” and “control,” the predicted risk of an event is higher for the “case”: values of 0.9–1.0 are considered outstanding, 0.8–0.9 excellent and 0.7–0.8 acceptable.^31^ In addition, we estimated the overall model performance^28^ using the Brier score (the average mean squared difference between predicted probabilities and actual outcomes, which also captures calibration and discrimination aspects^28^). Calibration (the agreement between observed outcomes and predictions^28^) was assessed using the regression slope of the prognostic index.^27,28^ Finally, since recent studies indicate that it is possible to achieve an unbiased and precise estimation of performance measures with a minimum of 100 events in the external validation data set,^32^ we also reported the number of events that were observed in the C&I NHS database.

#### Model Updating.

As a further exploratory step, we updated the model using the regression slope on prognostic index as shrinkage factor for recalibration, in line with the Royston et al guidelines.^30^

All analyses were conducted in STATA 11 and significance was set to *P* <.05.

## Results

### C&I NHS Trust Sample Characteristics

A total of 13702 patients accessing C&I NHS Trust between January 1, 2009 and October 30, 2016 received an ICD-10 index diagnosis other than psychosis. Patients accessing the C&I NHS Trust and included in the current study had an average age of 40 years (range 16–99, only 41 individuals were aged 16–17), 52% were female, and most were of white ethnicity (64%). The most frequent index diagnosis was nonbipolar mood disorders (28%). Missing data related mostly to ethnicity (10.8%, table 1).

**Table 1.** Sociodemographic Characteristics of the Camden and Islington (C&I) NHS Trust Compared With the South London and Maudsley (SLaM) NHS Trust, UK

Variable	C&I (*n* =13702)	SLaM (*n* = 33820)^a^	C&I Vs SLaM
	Mean (SD)	Mean (SD)	*P*
Age, y	40.91 (15.14)	34.4 (18.92)	<.001
	**No. (%**)	**No. (%**)	
Sex			<.001
Male	6582 (48.04)	17303 (51.16)	
Female	7118 (51.95)	16507 (48.81)	
Missing	2 (0.01)	10 (0.03)	
Ethnicity			<.001
Black	1189 (8.68)	6879 (20.34)	
White	8804 (64.25)	18627 (55.08)	
Asian	762 (5.56)	1129 (3.34)	
Mixed	469 (3.42)	1306 (3.86)	
Other	998 (7.28)	3466 (10.25)	
Missing	1480 (10.80)	2413 (7.13)	
Index diagnosis			<.001
CHR-P	—	314 (0.93)	
Acute and transient psychotic disorders	427 (2.74)	553 (1.64)	
Substance use disorders	3428 (25.04)	7149 (21.14)	
Bipolar mood disorders	936 (7.05)	950 (2.81)	
Nonbipolar mood disorders	3694 (27.70)	6302 (18.63)	
Anxiety disorders	3122 (22.50)	8235 (24.35)	
Personality disorders	1468 (10.45)	1286 (3.80)	
Developmental disorders	111 (0.80)	1412 (4.18)	
Childhood/adolescence onset disorders	295 (2.15)	4200 (12.42)	
Physiological syndromes	170 (1.25)	2555 (7.55)	
Mental retardation	51 (0.34)	864 (2.55)	

*Note:* Clinical High Risk State for psychosis (CHR-P) is defined on the basis of the At Risk Mental State criteria. The index diagnosis was formulated at baseline (time of the first contact with the NHS Trust).

^a^Derivation database: Lambeth and Southwark boroughs.

### Differences Between the C&I and SLaM NHS Trusts

#### Sociodemographic Differences.

As noted above, the C&I NHS Trust neither included CHR-P services nor child and adolescent services. As a result, the average age in the C&I NHS Trust was 6.5 years higher than in SLaM, whereas the proportion of developmental or childhood/adolescence onset disorders was lower in C&I than in SLaM (table 1, post hoc *P* < .001). There were also fewer males and considerably fewer patients of black ethnicity in the C&I Trust (8% vs 20%, post hoc *P* < .001). Compared to SLaM, substance abuse disorders, nonbipolar mood disorders, mood disorders, and personality disorders were more prevalent in the C&I NHS Trust, whereas anxiety disorders were relatively less frequent (post hoc *P* < .001). Finally, physiological syndromes were found to be more prevalent in SLaM as compared with the C&I NHS Trust (post hoc *P* < .001).

#### Cumulative Risk of Psychosis in C&I NHS Trust Compared With the SLaM NHS Trust.

**Fig. 3.**
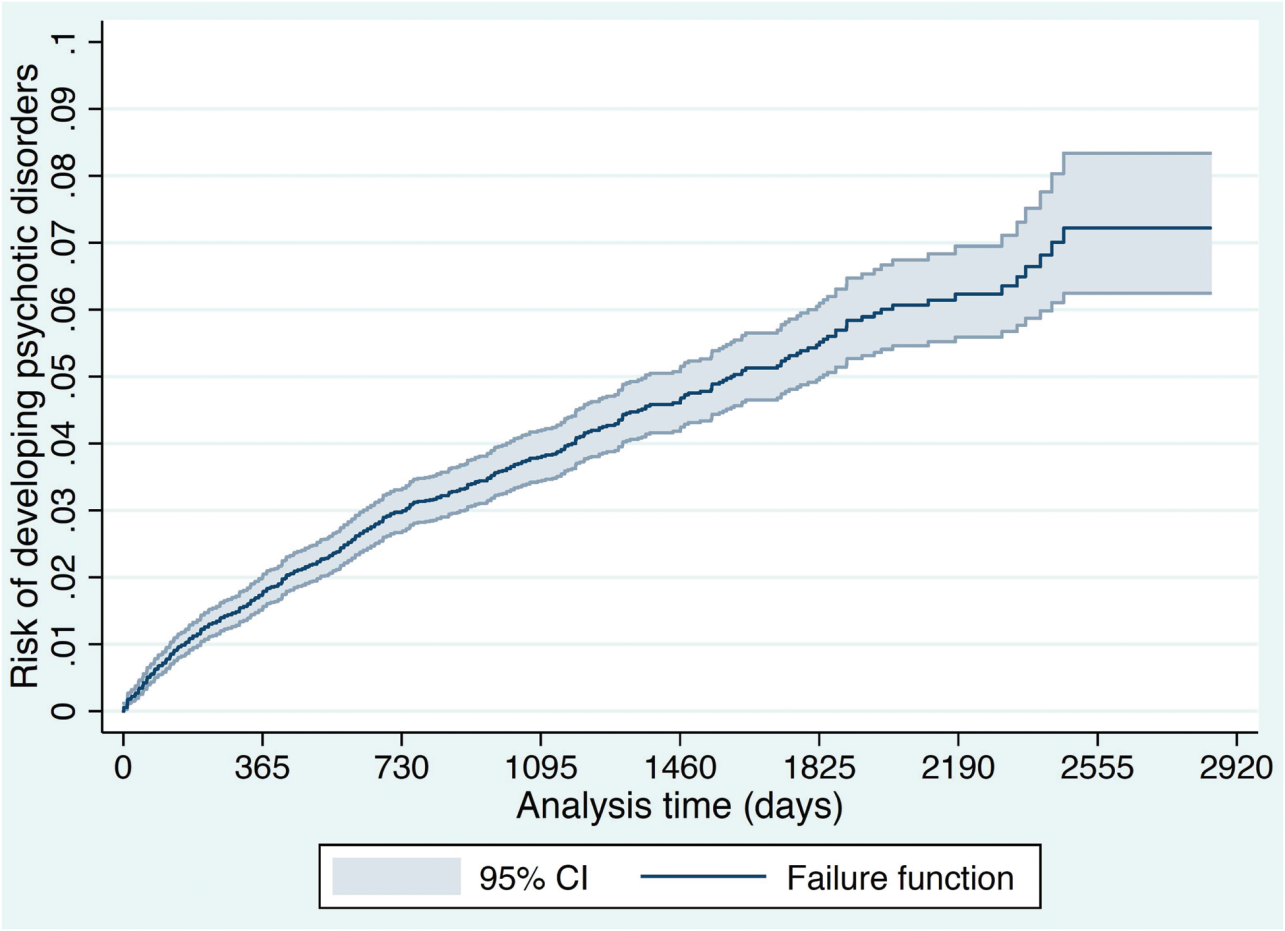
Cumulative incidence (Kaplan–Meier failure function) for risk of development of psychotic disorders in the Camden and Islington NHS Trust, UK. There were a total of 490 events (transition to psychosis). There were 212 events in the first 365 days, 123 events in the interval 365–730 days, 63 events in the interval 730–1095 days, 44 events in the interval 1095–1460 days, 28 events in the interval 1460–1825 days, 14 events in the interval 1825–2190 days, 6 events the interval 2190–2555 days, and no events in the interval 2555–2851 (end of follow-up). The last transition to psychosis was observed at 2466 days, when 13212 individuals were still at risk. The point estimates for cumulative incidence of psychosis were at: 1 year, 1.61; at 2 year, 2.76; 3 year, 3.53; 4 year, 4.36; 5 year, 5.19; and 6 year 5.88 (95% CI: 5.27–6.57).

The average follow-up time was 37.17 months (SD = 22.25). The average time to transition to psychosis was 20.08 months (SD = 18.14). The cumulative risk of psychosis in the C&I NHS Trust is plotted in figure 3, with the last transition being observed at 2466 days. The 6-year point estimate in SLaM was 3.02 (95% CI = 2.88–3.15), with the last transition being observed at 2099 days (see eFigure 1 in [ref^11^]). Mean values of the prognostic index within the C&I and SLaM Trusts were −1.06 and −1.32, respectively (post hoc *P* < .001). SD of the prognostic index in the C&I and SLaM Trusts were 0.84 and 0.89, respectively (post hoc *P* < .001).

### Second External Validation of the Original Model in the C&I NHS Trust

The primary performance measure in the C&I NHS Trust was acceptable, with a Harrell’s C of 0.73. The model was not well calibrated and was under-fitting the data, with a regression slope of 0.750, 95% CI = 0.687–0.813 (*P* < .001). The Brier score at 6-year was 0.038 (6-year Brier score in SLaM = 0.027). The full specifications of the model are reported in supplementary eTable 4.

### Model Updating

Model updating improved calibration (regression slope = 1), but there was no substantial improvement of model performance, which remained acceptable, with a significant Harrell’s C of 0.73.

## Discussion

This is one of the few predictive modeling replication studies in early psychosis. We showed evidence for large-scale clinical transportability of the transdiagnostic, clinically based, individualized risk calculator to another NHS Trust in the United Kingdom. The vast majority of predictors were available in the secondary NHS Trust and were collected as part of standard clinical practice. Compared with the NHS Trust wherein this model was developed, the secondary NHS Trust was characterized by significant differences in service configuration (there were no child/adolescent or CHR-P services) and in sociodemographic characteristics. In the new NHS Trust, the risk calculator was able to retain acceptable prognostic performance to a level comparable with that of current CHR-P psychometric interviews.

The key finding of this study is that the overall prognostic accuracy of the transdiagnostic clinically based risk calculator was externally replicated, resulting in acceptable performance statistics. Notably, the level of prognostic accuracy (Harrell’s C = 0.73) was comparable to that of CHR-P psychometric instruments currently used in clinical practice. In fact, our last prognostic meta-analysis showed that the Comprehensive Assessment of At Risk Mental States—which is the NICE-recommended instrument for detecting individuals at risk of psychosis—is characterized by similar adequate prognostic accuracy (area under the curve at 2 years = 0.79).^33^ These findings confirm the clinical utility of our transdiagnostic calculator, which has been estimated in our previous publication^13^ through rigorous decision curve analyses.^34^ Our risk calculator represents the only available pragmatic way to improve the detection of individuals at risk of psychosis in secondary mental health care.^12^ Systematically screening all individuals accessing NHS Trusts with widespread use of CHR-P tools is theoretically viable but logistically untenable and financially unsustainable. Implementing intensive outreach campaigns to promote referrals on clinicians’ suspicion of psychosis risk is highly inefficient and we have already demonstrated this issue at meta-analytical level.^11^ Intensive outreach campaigns lead to diluted risk enrichment^35^ and to negligible positive predictive values in those meeting CHR-P criteria.^11,36–38^ This second replication is therefore clinically important; as is the case in biomedical science more broadly,^39^ prognostic modeling in early psychosis suffers from a serious replication crisis,^40^ to the point that replication becomes equally as—or even more—important than discovery.^41^ A recent systematic review and meta-analysis of clinical prediction models for predicting the onset of psychosis in CHR-P samples uncovered 91 studies, none of which performed a true external validation of an existing model.^42^ Upon completion of this meta-analysis, another risk calculator was developed and validated externally,^43,44^ although its applicability is limited to individuals already meeting CHR-P criteria; it cannot, therefore, be utilized to boost the detection of at-risk individuals. In general, lack of replication is the primary barrier impeding the translation of research promises into real-life biomedical applications.^41^ In predictive modeling, this is often due to small samples,^45^ stepwise selection of variables based on significance threshold^46^ and the scarcity of events to be predicted.^32,42^ Our model successfully bypasses these caveats because it has been developed on robust a priori meta-analytical clinical knowledge, and validated in very large data sets (≈30000 patients) that encompassed many (400–1000) events.

Furthermore, this confirmatory result provides pragmatic support for the potential clinical transportability^19^ of our calculator into other NHS Trusts, at least within the United Kingdom. Demonstrating clinical validity requires evaluation of the predictive value in real world populations^39^ such as those encompassed by our real world, real-time electronic clinical registers. The significant (beyond chance) and acceptable prognostic performance is even more convincing in the context of the substantial differences found between the C&I and SLaM NHS Trusts. For one, the sociodemographic characteristics of the patients were dissimilar across Trusts, with SLaM being characterized by greater proportions of younger individuals and black ethnicity. More importantly, the C&I NHS Trust lacked child/adolescent mental health services as well as CHR-P services. These differences likely impacted the proportions of different ICD-10 index diagnoses across the 2 Trusts. In fact, within the C&I NHS Trust, greater proportions of substance abuse disorders, nonbipolar mood disorders, mood disorders, and personality disorders were observed. The mean and SD of the prognostic index across the 2 Trusts confirmed some degree of case mix, with higher predicted risks and less spread in the C&I Trust. Given such profound differences, it was expected that the risk calculator could not be easily transported to the local scenario, and thus a lower performance than that observed in the first external validation (Harrell’s C = 0.79)^13^ was expected. From a statistical point of view, the acceptable degree of difference between the development and external validation populations is a matter of debate.^30^ Successful replication is particularly relevant for recent national clinical guidelines and policies. NHS England and the Department of Health recently published the Access and Waiting Time Standard for Early Intervention and its NICE guidance.^47^ The guidance requires CHR-P individuals to be assessed and treated rapidly, and with evidence-based interventions.^47^ The NICE guidance in this area is not evidence-based and by making highly inefficient detection strategies a national priority, has set itself to failure. Not surprisingly, the NICE guidance is already introducing operational issues and concerns relating to unclear referral pathways and inefficient use of clinical resources.^48^ The efficient detection of at-risk cases marks the first step toward successful risk estimation tools for clinical practice.^49^ Our calculator can potentially fill such a translational gap, offering a pragmatic approach for the successful implementation of the NICE standard. This is particularly relevant for the majority of the UK-based NHS Trusts that lack established CHR-P services. Since the set-up of CHR-P services is challenging from both logistical and economic perspectives,^3,50^ we expect our calculator to play a pivotal role by optimizing the recruitment and referral strategies of available service configurations (see below).

The full implementation of this calculator in the wider clinical practice of the NHS clearly requires additional confirmatory evidence. The next steps would involve replication in further NHS Trusts in the United Kingdom and a prospective feasibility study. Our research team is currently conducting these studies. Upon completion of this research, we will seek to replicate our calculator outside the United Kingdom and will then conduct a definitive large-scale effectiveness trial to demonstrate its ultimate clinical utility in the real world. Four pragmatic aspects of our calculator may facilitate future research and its broad clinical use.^19^ First, the risk calculator is simple and only requires basic sociodemographic and clinical predictors. Indeed, missing data in the C&I NHS Trust were relatively rare,^13^ indicating that the calculator can possibly be used to test large numbers of cases. This is a key requisite to boost the detection power across the entire secondary mental health care sector. Our calculator was conceived and developed within an evidence-based pragmatic psychiatry approach.^51^ In a similar fashion, the best known, and probably the most widely used risk estimation tool used in medicine globally, is the Framingham pragmatic clinical prediction model. It includes simple variables such as sex, age, total and high-density lipoprotein (HDL) cholesterol, systolic blood pressure, smoking status, diabetes, and hypertensive treatment.^49^ Second, the risk calculator is cheap and does not involve costly processing, complex techniques, or other accompanying infrastructures. Rather, it increases the value of public money invested in training NHS clinicians, because it capitalizes on the time and psychopathological expertise already used by clinicians to formulate the index diagnoses. Risk estimation systems are of little value unless clinicians use them in day-to-day practice.^49^ Third, implementation of the risk calculator can leverage on e-Health apps and translational informatics, such as the electronic case registers of the National Institute for Health Research Biomedical Research Centres (NIHR-BRC), which have a presence not only in south London (SLaM), but also in other sites such as the C&I, Oxford, Cambridge, and Newcastle Upon Tyne NHS Trusts. In these sites, the risk calculator could be easily automatized, minimizing coding problems.^49^ At the same time, NHS Trusts that do not have an NIHR-BRC electronic case register could still use the online version, which has been made freely available.^13^ Fourth, the calculator is ageless, because its primary clinical aim is to detect all individuals at risk of developing psychosis at scale, in secondary mental health care. It can, therefore, be used in the 15–35 age range of peak psychosis risk^52^ as well as outside of this range. This is clinically important in the United Kingdom because with the recent introduction of new governmental acts,^53^ early intervention services for psychosis have become almost ageless.^54^

There are also some considerations for further research. This is the first transdiagnostic risk calculator available. Although the transdiagnostic approach is becoming popular, its exact meaning remains somewhat obscure. From the Latin etymology, the prefix “trans-” could mean either “across” or “beyond” (Oxford dictionary https://en.oxforddictionaries.com/definition/trans -). We here replicate—for the second time—the clinical reliability and usefulness of psychiatrists’ classification of broad diagnostic phenomenological spectra (the ICD-10 diagnoses were indeed pooled into larger diagnostic clusters as indicated in supplementary eTable 2) to build clinical prediction models. At a time of likely excessive claims and enthusiasm to abandon and go “beyond” psychiatric diagnoses, in the real world of busy NHS Trusts, classification of patients’ problems has yet to be replaced by anything better and is still entirely based on psychopathology.^55^ Our evidence-based pragmatic approach^51^ follows an incremental improvement of knowledge framework—which has worked in the rest of medicine—and thus rather than moving “beyond” broad diagnostic spectra, it works “across” them. Moreover, to be pragmatically useful, prognostic risk models must show above chance performance (ie, greater than 0.5), and if implemented on a large scale, even risk prediction models with a modest accuracy (of about 0.65) may be considered of clinical utility.^56^ Furthermore, the current model is based on a few predictors, and as such, it could represent a benchmark for future refinements. For example, neurobiological biomarkers that are currently under investigation hold potential promise for improving specificity.^57^ However, as more factors are included, the risk algorithm becomes more complex, time consuming, and costly. This increase in model complexity can impact the usage of the risk prediction tool in the broader clinical scenario.^49^ For a risk calculator to be pragmatically used in front-line clinical settings with the primary aim of improving the detection of individuals at risk of psychosis, sophisticated and cost-intensive neuroimaging or peripheral measures are unlikely to be a viable solution. Instead, our calculator could be used to primarily detect at-risk individuals as soon as they contact NHS secondary mental health services, as a first step. In the second step, those who are detected could undergo a proper CHR-P assessment. In a further step, those testing positive at the CHR-P interview could then be subjected to biomarker-based risk stratification models. In a recent meta-analytical simulation, we confirmed that such a sequential combination of clinical, electrophysiological, neuroimaging, and peripheral risk stratification models in CHR-P samples could potentially increase the prognostic accuracy.^58^ The general concept of sequential testing is in line with the clinical staging model of early psychosis^7^ and is an approach widely deployed in clinical medicine.

Limitations of this study are mostly inherited from the original model and are fully detailed in the supplementary eLimitations section. In brief, our diagnoses have high ecological but unclear psychometric validity, the research team carrying out this replication study is not completely independent from the original one^59^, it is possible that the model is charting out relationships that reflect diagnostic practice within the United Kingdom and randomized clinical trials or economic modeling are needed to assess whether our risk calculator effectively improves patient outcomes.

## Conclusions

The transdiagnostic risk calculator shows an acceptable performance even in NHS Trusts in the United Kingdom with different sociodemographic characteristics and service configurations. This calculator may support an improved detection of at-risk cases in secondary mental health care, as well as the transdiagnostic prediction of psychosis even in NHS Trust that do not provide CHR-P services.

## Funding

This study was supported by the King’s College London Confidence in Concept award from the Medical Research Council (MRC) (MC_PC_16048 to P.F.P).

## Supplementary Material

Supplementary Material
